# Rejecting the myth of equal opportunity: an agenda to eliminate racism in nursing education in the United States

**DOI:** 10.1186/s12912-021-00548-9

**Published:** 2021-02-09

**Authors:** Kechinyere C. Iheduru-Anderson, Monika M. Wahi

**Affiliations:** 1 The Herbert H. and Grace A. Dow College of Health Professions, CHP 2215, Central Michigan University, 48859 Mount Pleasant, US; 2 DethWench Professional Services, 30 Newbury Street, 3rd Floor, MA 02116 Boston, US

**Keywords:** Racism, Education, Nursing, Societies, Accreditation, Social Justice

## Abstract

**Background:**

Unfortunately, racism and discrimination against Ethnic minority (EM) has been globalized, universally infecting industries worldwide, and the field of nursing has not been spared. In the United States (US), overt and institutionalized racism (IR) still permeates the fields of nursing, nursing leadership, and nursing education. Programs to address these disparities, and efforts by nursing professional societies and nursing education policymaking bodies to address racism in the nursing field, specifically with nursing leadership and education, have met with little success.

**Objective:**

The purpose of this paper is to illustrate the existence and magnitude of racism and its impact on the fields of nursing, nursing leadership, and nursing education, and to make evidence-based recommendations for an agenda for reforming nursing education in the US.

**Methods:**

A narrative literature review was conducted with a focus on pulling together the strongest evidence on which to base policy recommendations.

**Results:**

Based on the available literature, we put forth five recommendations aimed at modifying nursing education in the US as a strategy to counter IR in the US in the nursing field.

**Conclusions:**

Recommendations to address IR in nursing focus on nursing education, and involve implementing programs to address the lack of opportunity for both EM students and faculty in nursing, developing an anti-discriminatory pedagogy, and incorporating diversity initiatives as key performance indicators (KPIs) in the process of approval and accreditation of nursing programs.

## Background

The problem of racism has persisted in the nursing field since its inception, and this has resulted in global and far-reaching implications [1]. Ethnic minority (EM) nurses continue to face discrimination in their roles in clinical care, but also experience inequity in the form of professional barriers to rising to the level of nursing leadership, or serving as nursing faculty[2]. Given the racism faced by EM nursing faculty and the generally racist environment in nursing, EM nursing students suffer from inequities in education. These features of institutionalized racism (IR) work together to perpetuate disparities against EM nurses, nurse leaders, nursing faculty, and nursing students, and complicate the role of nurses in promoting non-discriminatory, culturally-competent care and social justice with respect to their patients. The purpose of this paper is to illustrate the existence and magnitude of racism in its impact on the fields of nursing, nursing leadership, and nursing education, and to make evidence-based recommendations for an agenda for reforming nursing education in the United States (US).

## Methods

A narrative review was conducted, as it is appropriate to summarize the findings from a collection of studies that have used diverse methodologies and theoretical conceptualizations [3]. Articles included in this review were selected on the basis of the quality and utility of the evidence they provide [3].

### Definitions

Racism has been defined as, “A phenomena (sic) that results in avoidable and unfair inequalities in power, resources, and opportunities across racial or ethnic groups” [4]. Racism can be expressed in different ways, including, “Through beliefs (e.g., negative and inaccurate stereotypes), emotions (e.g., fear/hatred), or behaviors/practices (e.g., unfair treatment), ranging from open threats and insults (including physical violence) to phenomena deeply embedded in social systems and structures” [4]. Racism has also been described as institutional or individual practices that create a system by which racial or ethnic groups are continually oppressed [5].

Race and ethnicity, therefore, refer to socially-constructed (rather than biologically-constructed) groups who often share the same cultural heritage and ancestry [5]. These systems of socially-constructed race and ethnicity groups are actually forged by oppressive systems created by majority groups using ideology in which the story explains why the majority group should benefit from dominating over the other groups, and should continue to define itself as well as the other groups through this domination relationship [5]. Further, these systems continue to define these oppressed racial and ethnic groups by way of possession of selective and arbitrary physical characteristics (most notably skin color) [5]. It follows, then, that discrimination against EM refers to the process by which members of these defined, oppressed racial and ethnic groups are treated differently (usually unfairly) simply due to their believed membership in the group [5].

Although a decline has been observed in overt racist attitudes in contemporary society due to social unacceptability, it is unclear to what extent actual individuals still believe in racial stereotypes or ideologies [6]. Because of this, a discussion of IR cannot be complete without a discussion of white privilege [7, 8]. According to DiAngelo [7], it is normal for common discourse to include racially-coded language, such as “urban,” “inner city,” and “disadvantaged” when talking about black people, but not using the terms “white”, “overadvantaged” or “privileged” when talking about white people (p. 55). She asserts that this coded language reproduces racist images and perceptions while it simultaneously perpetuates the comfortable illusion that race, and problems associated with race, are the fault of EM [7].

Liu [9] discussed the formation of privileged identities, using the following definition of privilege: “a multi-identity act that is facilitated and supported by institutions and organizations” (p. 349). These institutions act as “power-governors” that function to support an ideology of white supremacy (p. 349), and this way, actors within the system function to perpetuate and legitimize this inequity so that it is maintained as the status quo [9]. IR is related to the concept of implicit bias, which is a belief that is triggered unconsciously that holds that a particular group is inferior, or otherwise sees a particular group with a negative attitude [6].

## Results & discussion

Both overt racism and IR have impacted the field of nursing in many different ways. The impact of racism in nursing extends to nursing leadership and nursing education, and ultimately results in negative consequences for patients who deserve culturally-competent and non-discriminatory care. This section will explain how this complex system of both overt racism and IR has worked to integrate racism into the very foundations of nursing, nursing leadership, and nursing education.

### Racism in Nursing

Regrettably, racial discrimination against EM happens in all countries, universally impacting all sectors, without sparing the field of nursing [1, 10]. As an illustration of this, although as of July 1, 2017, EM made up over 38 % of the United States (US) population [11], findings from the 2017 National Council of State Boards of Nursing (NCSBN) Workforce Survey, which includes US nurses, showed that only 19.3 % of registered nurses (RN) identified as EM [12].

This phenomenon is not only present in the US. In the United Kingdom (UK), The Royal College of Nursing (RCN) acknowledged that IR is a problem in UK nursing [13]. Studies in both the US and the UK have revealed pay disparities, in that the average hourly wage for black RNs is lower when compared to white RNs [14–17]. The Journal of Nursing Management (JNM) conducted a survey in 2010, and included samples of nurses from the US, Saudi Arabia, Canada, China, and New Zealand [18]. They found that among nurses who reported making over $120,000 annually, over 90 % were Caucasian [18]. By contrast, this highest income stratum only included 4 % of black nurses, and 2 % of Hispanic or Asian nurses [18].

An interview study was conducted in the UK among nursing managers who were asked about their experiences with black African nurses [19]. On one hand, the managers described how they observed these nurses being treated in racist ways by both patients and colleagues [19]. But the managers also reported that these nurses suffered from racism that was coming from the job itself, such as not being provided equal opportunities for and experiencing discrimination from colleagues and managers [19].

The issue of racism directed at EM nurses from many different sources is particularly apparent when considering internationally-educated nurses (IEN), or nurses who originate in non-Western countries and transition to Western workplaces, such as those in the UK, US, Canada or Australia [20, 21]. One interview study of Chinese IENs transitioning to the US found several main themes, including “injustice and discrimination” coming from many different sources, including patients, colleagues, managers, and employers [22]. A study from the UK also described this phenomenon of injustice and discrimination directed at EM nurses from multiple sources, and called it “racist bullying” [23]. A different study examining barriers and facilitators for EINs successfully transitioning to a Western work setting found that a common barrier was racism, and a common facilitator was developing skills to counter racism [24].

Braithwaite has described how IR has been institutionalized in the nursing field as part of an extension of colonialism [25]. The 1960s was a period of migration, where EM female nurses moved to the UK and former UK colonies in search of work [25]. Over time, the overt racism these women initially experienced decreased, but this colonialism was still embedded in the foundation, and this colonialism continues to present itself in the form of disparities in how white and EM nurses are treated in the UK [25]. In this way, Braithwaite argues that modern day UK nursing remains colonized, and EM nurses are modern day victims as they struggle against racial stereotypes and institutions that continue to rob them of power [25]. Although the history of colonization in the US is different, the US has a similar problem as the UK, where African slavery and other historical events have embedded racism into the foundations of the healthcare system, thus continuing historical racism and health inequities with respect to EM nurses and patients [26].

Barbee wrote a landmark paper in 1993 that focuses specifically on why racism has been perpetrated in US nursing [27]. She highlights four attributes of nursing that inadvertently create weaknesses in the field, making it susceptible to be co-opted with racist ideas [27]. First, nursing emphasizes empathy, and this leads to the corollary that all patients should be treated equally without consideration of race [27]. This attribute creates the unintended barrier of nurses being uncomfortable to admit racism is in the field, because that suggests the operational practice of empathy in nursing may need reform. Second, nursing emphasizes an individual orientation, which encourages taking focus off of structures in society that might be a source of influence, such as social norms and political climate [27]. This same principle that encourages nurses to treat the poor and the rich equally also has the unintended effect of leading nurses to turn a blind eye to a patient’s socioeconomic status. This tendency distracts nurses away from perceiving the intersection between race and socioeconomic status, and from seeing the connection between racism and poverty in their patients [27].

Third, in order to develop efficient protocols for delivering nursing services and education, the nursing field prefers homogeneity with respect to both patients and students [27]. Favoring homogenous patient and nurse pools as part of structuring and delivering nursing services and education runs counter to the real level of diversity with respect to the wide variation among actual patients and nurses [27]. Hence, this preference operationally is translated into resistance to accommodating this real diversity in both patient and nurse populations [27]. Finally, the nursing field emphasizes a desire to maintain a peaceful climate in the workplace by avoiding conflict [27]. While this seems reasonable, it inadvertently presents resistance to other nursing functions, such as identifying and addressing safety problems, or other issues that might cause workplace conflict as an outcome of legitimate business processes [27]. The consequence of the principle of emphasizing conflict avoidance essentially provides cover to those in the nursing field, especially leaders, who want to avoid openly addressing the scourge of racism in the workplace under the guise of maintaining a peaceful climate [27].

### Racism in Nursing Leadership

Racism in the nursing field has impacted the demographics of nursing leadership over time. The Institute for Diversity in Health Management (IDHM), which is an affiliate of the American Hospital Association (AHA), conducted a national survey in 2015 of over 6,000 chief executive officers (CEOs) of hospitals in the US [28]. The survey found that only 14 % of hospital boards were comprised of EM, only 11 % of hospital executive leaders were EM, and among first and midlevel managers, only 19 % were EM [28]. This lack of EM at leadership levels prompted the US National Academy of Medicine (NAM), formerly called the Institute of Medicine (IOM), to make a call for efforts to increase EM representation in nursing leadership in their 2010 report [29]. The results of NCSBN Workforce Survey in 2015 that was mentioned earlier revealed that 14.6 % of black or African American nurses in the US have nursing-associated masters or doctoral degrees compared to 13.4 % of white nurses, so there exists a qualified pool of potential EM leadership candidates [30].

The UK also struggles with this problem. As reported in the Health Service Journal in 2016, a panel discussion was held to discuss the problem of lack of EM among the ranks of nursing leadership in the UK [31]. The panel concluded that the National Health Service (NHS) trust boards failed with respect to their duty to promote EM representation in nursing leadership [31]. As evidence, they cited a decrease in black people appointed to NHS trust boards from a high of 9.6 % in 2006 to a decrease to 8 % in 2014 [31]. EM staff in the NHS have been found to have fewer opportunities for development and career progression, so a range of initiatives and interventions were deployed by the NHS to address this [13, 32]. Yet, as shown by the results of the discussion panel, these initiatives, which were implemented during a stretch of time that included the period between 2006 and 2014, did not increase EM participation in nursing leadership. In fact, over this period of time, EM participation actually decreased.

Ironically, in the study mentioned earlier about the observations of nursing managers in the UK of racism directed at black African nurses, the managers themselves at times expressed racist views [19]. Specifically, the managers reported that they perceived that black African nurses lacked motivation to pursue promotions or professional development [19]. This situation presents a unique challenge for efforts aimed at addressing racism in nursing leadership. Since EM RNs report barriers to promotional and development opportunities more frequently than their white counterparts, this characterization of the issue as “lack of motivation” when the real issue is “lack of opportunity” illustrates a potentially vicious cycle [2, 20, 33].

The lack of EM represented among nursing leadership has several implications. First, it is challenging to conceive of being able to provide “culturally competent” care within a framework of a field where underlying issues of racism and discrimination have not yet been adequately examined and confronted [34]. Although cultural competence in nursing is admittedly ill-defined, it roughly translates to the idea of being openminded to ethnic and cultural diversity among patients, and working to accommodate these differences when delivering nursing care [35, 36]. Nurses are ostensibly expected to deliver so-called culturally competent care, but this very mandate is problematic when called for within a field where racism is pervasive and relatively unchecked [17]. Lack of culturally competent care has been identified as one of the causes leading to health disparities in patients, so this inattention to structural racism in the nursing field results in barriers to its ability to confront the negative impact of racism on patient health [17]. Second, the situation where nurse leaders believe that EM nurses lack motivation for career development, when in reality they are being actively blocked from advancement, represents a toxic barrier to addressing IR in nursing [37–39]. Third, the barriers to EM leadership in nursing result in a lack of EM role models in advanced positions in nursing [40]. As mentoring and role modeling are critical to the transfer of knowledge in nursing, lack of EM role models in nursing compromises the opportunity for professional development among EM nurses in clinical care and nursing leadership as well as academia [40].

### Racism in Nursing Education

IR causes challenges for EM in all areas of academia, not only nursing. One study showed the rate of EM faculty in general is often disproportionately low, and that once EM faculty are hired, they are typically subjected to both subtle and overt racism, are the subjects of stereotypes and racist assumptions, and are marginalized [41]. Further, EM faculty often find their research discredited, especially if it is on a topic of specific relevance to EM populations, and are treated with tokenism [41]. In a grounded theory study conducted by Hassouneh and colleagues, it was found that non-EM faculty employed strategies of exclusion and control aimed at preventing EM faculty from having equal access to workplace opportunities [37]. Another study of African American nursing faculty found similar themes, including lack of clarity in job expectations, lack of job security, lack of diversity in the workplace, and an experience of racism [38].

Another issue in nursing and nursing education is that the educational content itself has problems with racism. Studies of education in medical professionals have found that learning experiences tend to be rife with racial bias [42, 43]. Although race is a social construct, it is often taught in health professions as a biologic feature, and this misunderstanding promotes biased thinking among clinicians [42]. Teaching about racism in nursing is another challenge, as one study of white nursing faculty found that their whiteness obscured their understanding of race, and therefore, they were not well-prepared to teach about race and racism [44]. A different study of UK nurse lecturers found that these educators were unconfident about their own abilities to teach about culture and racism, and address those topics in their curricula [45]. On the other hand, research shows that appropriating EM faculty to teach about racial concepts is not a viable solution, because they risk being tokenized [37, 41].

Another reflection of racism in nursing education is seen in the lack of faculty diversity. In the 2013 NCSBN workforce survey, it was found that EM nurses were underrepresented in nursing education at the faculty and administrative levels [46]. According to estimates by the American Association of Colleges of Nursing (AACN), the percentage of nursing faculty that were EM in the US overall was 16 % in 2016, and specifically for full professors, it was only 10 % [47]. The National League for Nursing’s (NLN’s) 2017 Faculty Census Survey found that over 80.9 % of nursing faculty were white, and African Americans only made up 8.8 % of full-time nursing educators [48]. Because there are so few EM among the ranks of the nursing faculty, these EM tend to have experiences as a “token” or a “lone ranger”, and not treated with equal status [49]. Hassouneh explains how these are manifestations of unconscious racist bias that serve as a barrier to developing a diverse nursing faculty, and provides examples that include inadequate career developing pipelines; disproportionately rare opportunities with respect to appointment, promotion, and tenure; and an unwelcoming academic environment overall [50].

Racism also taints the delivery of nursing education. A focus group study by Tilki and colleagues aimed to understand the experiences of racism in nursing education by students and lecturers [51]. Although overt racism was not routinely apparent, there was clear evidence of IR through Eurocentric values embedded in organizational culture and practices [51]. As an example, despite the fact that ethnically diverse students were prevalent in the educational setting, they were still considered the “other” [51]. A different study focusing on African American nursing students reported that most participants stated they faced racial discrimination in education [14]. These repeated reports of nursing students experiencing racism in their educational settings exemplifies the weak institutional commitments made by nursing colleges to students, and especially EM students [14].

This weak institutional commitment is also evident in studies that show that EM students face barriers in nursing learning environments [52]. These barriers could be seen as falling in two categories: those directly related to the student’s personal circumstances, and those relating directly to the nursing faculty and nursing education learning environment [52]. The lack of institutional support of EM students in nursing schools has a profound impact on these students. In 2016, the NLN published a report titled “Achieving Diversity and Meaningful Inclusion in Nursing Education,” where the authors emphasized that nursing pipeline programs are the key to improving representation of EM students in health professions [53]. This is relatively aspirational in the current environment, where one study found that only 20 % of nursing schools had a structured diversity pipeline program [54].

Racism impacts nursing education at all levels, and this has two particularly important implications. First, because nursing students are acquiring their skills in the setting of a racist educational environment, they will not believe to develop aptitude in delivering culturally-competent care [34]. Second, as the US healthcare system is rife with well-documented racial health disparities, ironically, nursing has been promoted as the professional field tasked with addressing this [55]. Given the racism inherent in the nursing field at all levels including education, it is difficult to imagine US nursing adequately addressing the pervasive problem of racial health disparities in the US healthcare system.

### Programs to increase diversity in Nursing Education

Programs exist to increase EM diversity in nursing education that are aimed at students as well as faculty [56]. It is possible to measure the climate of IR and how welcoming the workplace environment is to EM at both the student and faculty level [57]. One set of authors recommend teaching nursing students a “code of conduct” that could lead to inclusion and diversity in their patients; this could be adopted as a code of conduct to apply to entire nursing programs, including all the professionals and students in the program [58].

In terms of students, because EM students may have features that make them require extra support, interventions aimed at empowering or otherwise intervening on ethnic minority nursing students have been proposed [59]. These programs may be aimed at recruitment, retention, or both [60, 61]. However, these programs often have so many barriers to participation that EM nursing students cannot benefit from them. One example in the Robert Wood Johnson Foundation (RWJF) Future of Nursing Scholarship Program [62]. The program provides scholarships, mentoring, leadership development opportunities, and when applicable, post-doctoral research support to EM nurses for the purposes of building leadership capacity [62]. The program provides monetary support for EM nurses seeking doctoral education as well as one-year of competitive post-doctoral support [62]. However, there is a lot of effort involved in applying for this program and participating in it, and many EM nurses do not have the resources or ability to participate in such an intensive program. Further, it is limited to nursing doctoral students.

For faculty, programs can include leadership and mentoring programs. For example, the University of Tennessee Health Science Center (UTHSC) College of Nursing developed a Minority Faculty Fellowship Program aimed at developing and promoting Hispanic faculty, thus addressing the pipeline problem [63]. Again, these programs face the problem that EM nurses, nursing leaders, and nursing faculty are already suffering from racism in terms of less advancement, lower pay, and lack of opportunity. One example program is from the Minority Nurse Leadership Institute (MNLI) at Rutgers University, which offers a 10-month mentored leadership development curriculum [64]. Participants actively participate in ten Saturday seminars over the school year, meet monthly with a leadership mentor, and complete a team project designed to improve evidence-based nursing practice [64]. While this program seems laudable, there are often barriers to participation at this level for EM nurses, who already face lower pay at their jobs and may not have extra time to participate. Further, participation in programs such as these often needs to be approved or otherwise supported by the EM nurse’s advisor or supervisor at work, and that can pose an opportunity for control strategies to be used to prevent the EM nurse from participating [37].

### Response from professional societies

Professional nursing organizations have attempted to address the problem of racism in nursing in different ways. The American Nurses Association (ANA) published a position statement in 2018 about the nurse’s role in addressing discrimination, but it was very general and did not make any recommendations for dealing with the root causes of racism in nursing [65]. The International Council of Nurses (ICN) also does not address the issue direction. The closest position statement the ICN has to the topic of eliminating racism in nursing is the one addressing cultural and linguistic competence, which again does not examine how to deal with the root causes of racism in nursing [66].

While EM professional nursing organizations like National Black Nurses Association (NBNA) play an important role in the elevation of EM nurses to leadership, they cannot be tasked with eliminating racism in nursing [67]. As was shown by the research presented earlier, many non-EM nursing educators do not feel prepared to teach cultural and race issues in nursing, yet, nursing education still holds the goal of teaching “cultural competence” [34].

### Governance of Nursing Education in the United States

In the US, in order to obtain a nursing license to practice, one must pass the National Council Licensure Examination-Registered Nurse Examination (NCLEX), but in order to qualify to sit for the NCLEX, the individual must graduate from a nursing program approved by the state-level board of nursing (BON) of the state in which the nursing program resides [68]. For this reason, BONs must stay synchronized with respect to educational standards such that across the US, approved nursing programs perform at least minimally to the point that an acceptable proportion of their students are able to pass the NCLEX [68]. This synchronization is facilitated by the participation of the BON in the NCSBN [68]. Currently, there are no NCSBN requirements about EM nursing students or faculty that are part of criteria for approval of a nursing program [68].

Approved nursing programs in the US may choose to seek accreditation, which is a non-governmental, voluntary, self-regulatory and peer-review process by which to recognize educational nursing programs that meet or exceed standards and criteria set to ensure high educational quality [69]. The three nursing program accreditation bodies in the US are the Accreditation Commission for Education in Nursing (ACEN) [69], the American AACN’s Commission on Collegiate Nursing Education (CCNE) [70], and the NLN’s Commission for Nursing Education Accreditation (CNEA) [71]. Each of these programs has a slightly different set of standards, but all of them have criteria relating to mission, faculty resources, student resources, and program outcomes [69–71]. Of these three, only the CNEA has criteria that relates to EM diversity and inclusion with respect to both students and faculty [71].

Although up to now, state BONs, the NCSBN, and the accreditation bodies have not directly taken efforts to confront the scourge of racism integrated throughout the nursing field and negatively impacting nursing education, the NLN released a report in 2016 with a vision to achieve diversity and meaningful including in nursing education [53]. Although this living document provides a vision, currently, no part of the nursing education approval or accreditation infrastructure mandates any criteria with respect to the inclusion and treatment of EM as nursing students or nursing faculty.

## Recommendations

Unfortunately, racism is currently embedded in the nursing field, and negatively impacts nursing leadership and education such that EM nursing students and nurses are continuously disadvantaged. In the US, the NCSBN conducts regular surveys to quantify the demographics, occupational status, and other characteristics of those in the nursing field. Their efforts provide quantitative data that can be seen as benchmarks measuring the magnitude of how racism is impacting nursing in the US. The NCSBN, working in conjunction with the accreditation bodies and state lawmakers, can facilitate incorporating new criteria into both approval and accreditation of nursing programs that can shape nursing education policy to improve support for EM nursing students and faculty, and the development of leadership skills in EM nurses. Experience has shown that holding organizations accountable to policies is necessary for addressing diversity issues in the workplace [72]. Further, nursing accreditation agencies have tried to play a role promoting diversity in academic nursing, but it likely would be much more effective if they incorporated diversity standards into their accreditation criteria [73].

Considering this, we make five recommendations, as shown in Table 1.

**Table 1:** Recommendations for Improving Nursing Education for Ethnic Minorities in the US

Recommendation	Audience	Level	Intended Results
**Mandate Nursing Programs to Meet EM Student Benchmarks**: Unless mandates are established to compel nursing colleges to modify nursing education to specifically target educational and leadership advancement for EM nursing students and require them to report their progress in key performance indicators (KPIs) in order to remain accredited, there will be likely no improvement in the quality of EM nursing education and in EM nursing leadership advancement.	Nursing Education Administrators, Nursing Professional Societies and Nursing Program Accreditation Boards	Policy	• Increase the quality of nursing education and opportunities to build leadership skills in EM nursing students • Position statements on EM distribution should be revised and linked directly to KPIs • KPI’s could consider EM student transfers out of the program, EM student grade levels compared to other students, and other measures of EM student success
**Mandate Nursing Programs to Meet EM Faculty Benchmarks**: As part of accreditation KPIs, require a set percentage of EM nursing faculty and EM nursing faculty leaders, and source these professionals from programs from EM nursing professional societies established for the express purpose of helping colleges fulfill this mandate.	Nursing Education Administrators, Nursing Professional Societies and Nursing Program Accreditation Boards	Policy and Community	• Increase EM nursing faculty and EM nursing faculty leadership • Provide a framework for establishing succession planning to maintain level of EM participation • Ensure that initiatives aimed at increasing EM nursing faculty and faculty leadership in nursing programs are not appropriated to particular job classes, titles, or sections, but are enacted program-wide • KPIs could consider percentage of EM faculty and faculty leaders, EM faculty and leadership attrition rates, EM faculty pay parity, as well as other metrics
**Empower Nursing Faculty with New Skills**: Teach nursing faculty skills to counteract overt, covert and institutionalized racism in nursing education.	Nursing Educators and Nurse Education Leaders	Individual and Community	• Reduce discomfort in non-EM nursing educators in discussing white privilege and issues associated with institutionalized racism • Prevent tokenism and lone ranger status to EM faculty and students • Improve the quality of nursing education overall
**Reduce Barriers to EM Nursing Students**: Reduce barriers to access educational programs aimed at increasing educational quality and leadership skills in EM nursing students.	Nursing Education Administrators and Nursing Professional Societies	Community	Increase the pool of qualified EM nursing applicants to nursing positions in all fields, including education and leadership
**Nursing Boards to Conduct Surveillance**: Maintain national annual surveillance for key metrics associated with improving advancement for EM in nursing, nursing leadership, and nursing education	National Council of State Boards of Nursing (NCSBN)	Policy	• Ensure that a central regulatory body (such as the NCSBN) conducts or supervises the conduct of routine surveillance to measure these metrics • Have the NCSBN establish and promote a minimal dataset of questions to be asked on state licensing applications that can be used for benchmarking progress annually toward EM-related initiatives

This section will describe these recommendations and how they are intended to counteract racism in the nursing field in the US.

### Recommendation #1: Mandate Nursing Programs to Meet EM Student Benchmarks

In their role organizing BONs, the NCBSN is in the perfect position to require nursing programs to reach EM student benchmarks in order to be approved. The NCBSN should not require merely the measurement of the number of EM students in the program, but should develop key performance indicators (KPIs) that not only speak to the participation of EM students in the program, but measure the level of success they have. If the NCBSN declined to allow BONs to approve of programs that did not meet these KPIs, it would go a long way toward addressing racism in nursing education in the US.

### Recommendation #2: Mandate Nursing Programs to Meet EM Faculty Benchmarks

In addition to mandating that BONs set up KPIs that must be met with respect to nursing programs serving EM students, the NCBSN could develop KPIs for EM nursing faculty in the program as well. KPIs would not only include the proportion of EM nursing faculty, as well as proportion of EM nursing faculty in leadership positions, but they would also speak to the quality of EM faculty development available in the nursing program, and the success of the program’s efforts to promote EM nursing faculty.

Practical regulatory implications of BONs using KPIs related to benchmarks associated with proportions of EM nursing students and faculty will admittedly come under scrutiny in the US, where efforts at increasing EM participation in academia are often met with legal challenges [74]. Nevertheless, reasonable goals could be set with regard to EM-related KPIs by BONs. The question arises as to how to address nursing programs that do not meet those goals. Putting such programs on “probation” and providing them BON-sponsored technical assistance to enable them to make progress toward the EM-related KPIs represents one politically feasible response that facilitates reform. Responses will necessarily be state- and program-specific, but should be aimed at promoting reform of the nursing program when it does not meet BON-mandated KPIs.

### Recommendation #3: empower Nursing Faculty with New skills

In 2017, the Research in Medical Education (RIME) Program Planning Committee developed a call for studies on racism and bias in health professional education so that recommendations could be developed to counter it [42]. Among the focuses of the Committee was to consider three roles – educator, faculty developer, and researcher – and recommend research questions to these roles that could be answered with studies that would provide evidence on which to base improvements [42]. One area of research focus recommended was on improving curricula and faculty instruction so that it was more inclusive and less biased [42].

One project implemented at the University of Washington School of Nursing worked to change the climate of whiteness in academic nursing by providing faculty workshops and teaching not only didactic information about whiteness and IR, but also, how to counter against it in the process of educational delivery [43]. Allen and colleagues [75] made the connection between anti-discriminatory teaching and the teaching of cross-cultural and culturally-competent care, and worked to promote an anti-discriminatory and cross-cultural curriculum at their Australian nursing program. Although Hassouneh [76] identified many challenges faced by EM faculty in implementing an anti-racist pedagogy in nursing, if developing and implementing an anti-racist pedagogy is the responsibility of nursing education leadership, this should take the pressure off of individual EM faculty.

### Recommendation #4: reduce barriers to EM Nursing students

Not only do EM students face barriers to participation in nursing education due to racism, they also often face barriers to participation in the very programs designed to counter the effects of racism and provide support to EM nursing students. For this reason, designers of such programs should strongly consider the cumulative impact that racism may have had on the EM nursing student prior to them entering a nursing education program. The lifelong impact of racism may have left them with less in the way of financial and time resources to devote to their nursing education in the first place. Also, as described earlier, nursing programs have racist features that further negatively impact EM students. Thus, adding support resources external to EM student’s nursing educational program may risk overloading them. Any efforts intended to support EM nursing students should focus on finding ways to offer individual empowerment to the student so that they may benefit maximally from their educational opportunities, and avoid requiring the student to invest additional time and financial resources they may not have to spare.

### Recommendation #5: BONs to Conduct Surveillance

Although occasional surveys are being done by places like the IDHM, finding the exact number of EM nurses in leadership positions is very challenging, because there is no standard for collecting and reporting data across institutions and organizations [28]. This surveillance could be incorporated into the NCSBN survey, which would be logical, because then all of the KPIs proposed in new policy would be measured reliably. This would facilitate fair processes associated with program approval and accreditation, as the NCSBN is seen as a reliable and valid survey in the nursing profession.

## Conclusions

In conclusion, this paper sets forth an agenda with evidence-based recommendations for reforming nursing education in the US to directly counter both overt racism and IR in nursing, nursing leadership, and nursing education. Because racism is embedded in the field of nursing and efforts to remove it have shown it is recalcitrant, without the active intervention of policymakers at the national level, likely, no progress can be made in this area.

## Data Availability

Not applicable.

## Abbreviations

AACN: American Association of Colleges of Nursing
AHA: American Hospital Association
ANA: American Nurses Association
CCNE: Commission on Collegiate Nursing Education
CEOs: Chief executive officers
CNEA: Commission for Nursing Education Accreditation
EM: Ethnic minority
ICN: International Council of Nurses
IDHM: Institute for Diversity in Health Management
IEN: Internationally-educated nurse
IR: Institutionalized racism
KPI: Key performance indicators
NAM: National Academy of Medicine
NBNA: National Black Nurses Association
NCLEX: National Council Licensure Examination
NCSBN: National Council of State Boards of Nursing
NHS: National Health Service
NLN: National League for Nursing
RCN: Royal College of Nursing
RIME: Research in Medical Education
RN: Registered nurses
RWJF: Robert Wood Johnson Foundation
UK: United Kingdom
US: United States
